# A model for lifetime analysis – application to clinical laboratory instruments using computer facilities

**DOI:** 10.1155/S1463924687000361

**Published:** 1987

**Authors:** F. R. Hindriks, W. Liet, A. Bosman

**Affiliations:** 1 Central Laboratory for Clinical Chemistry University Hospital Groningen Postbox 30.001 Groningen 9700 RB The Netherlands; 2 Department of Management Science and Business Administration of the Economic Faculty University of Groningen Groningen The Netherlands


Journal of Automatic Chemistry/*Journal of Clinical Laboratory Automation*, Vol. 9, No. 4 (October–December 1987), pp. 184–188

### A model for lifetime analysis – application to clinical laboratory instruments using computer facilities

**F. R. Hindriks,**
*Central Laboratory for Clinical Chemistry, University Hospital Groningen, Groningen, The Netherlands*

**W. Liet and A. Bosman**
*Department of Management Science and Business Administration of the Economic Faculty, University of Groningen, Groningen, The Netherlands*

*An investment model for the lifetime analysis of capital investments, for example instruments, in the clinical chemistry laboratory has been developed. All costs have been considered in relation to the actual time they were incurred. Based on the criterion of minimal costs for clinical chemistry tests, the economic lifetime of investments can be estimated. The model described can be an important tool in laboratory management and a great help in discussions about the allocation of financial resources. The model is applicable to every laboratory.*

### Introduction

A lot of attention is paid today to the cost of clinical chemistry tests. The policy-makers in the health services, the health insurance companies and the managers and directors of hospitals and institutions are regularly cited by the news media about their concern with regard to the inflation in the costs of, among other things, laboratory tests. Many studies have been published on the question of the cost of clinical chemistry tests [1–5]. However, in those studies the question of determining the economic lifetime of investments is not often dealt with in any detail. In practice such investments are included in the cost of clinical chemistry tests without analysing the economic lifetime.

In the present paper the financial basis for purchasing clinical chemistry instruments is determined in a generally applicable investment model. On the grounds of *prospective data* and the application of the criterion of *minimal costs,* the lifetime of an analysis instrument, which one might wish to purchase, can be estimated. The results of the model can be adjusted in *retrospect* to the actual cost of developments. In this model the cost at particular times can be compared using the well-known – in the field of business administration – discounting method [6 and 7].

### Investment selection, lifetime analysis and depreciation

Basically, the problem of investing in *fixed assets* is no different from investing in *floating means of production.*

Correspondence to Dr F. R. Hindriks, Central Laboratory for Clinical Chemistry, University Hospital Groningen, Postbox 30.001, 9700 RB Groningen, The Netherlands.

Fixed assets (durable means of production) should be interpreted as assets, whereby the application can be attributed to successive production processes. One of the most difficult problems in ascribing the fixed assets to several production processes is the fact that depreciation of the assets often does not lie in an objectively demonstrable relationship with the application in one production process. Deduced from the method of application, the investment and other expenses of the fixed assets are divided over successive production processes and usually over several years.

There are a number of reasons why investing in fixed assets differs from investing in floating assets. In the first place, time plays a much greater part in investments in durable means of production (fixed assets) than it does in floating means of production. Secondly, there are production/technical connections of means of production. Durable means of production will generally only be productive in combination with other production factors, such as labour and raw materials. These production factors should be considered as a conglomeration (unit) and not as a random collection. The achievements of durable means of production are therefore considered to be part of a unit. In the selection of investments an estimation is made beforehand of the possible combinations of production factors which will result in the lowest cost over the total lifetime. It is, of course, logical that the price per unit product in this context is considered over the whole lifetime.

In an investment project it is necessary to consider the total investment made in associated durable means of production and all additional investments in floating assets (such as, for example, stocks of reagents and disposables).

Principles from business management, for example the average accountable productiveness, the return-on-investment period and the internal productiveness, are used to choose the most profitable investment project and are not applied in the determination of the economic lifetime of a durable means of production. They are often used in connection with the economic lifetime, but do not form a judgement about it. This often gives rise to confusion.

The technical lifetime of an analysis instrument is the period of time that the instrument is technically capable of conducting the activities for which it was purchased. In determining the economic lifetime, the deciding factor is the period of time over which it is considered to be economically worthwhile to use the instrument. The economic lifetime of a durable means of production is determined via various factors, partly by factors which are inherent to technical functioning (wear and servicing) and partly by the economic and technical circumstances (for example changes in the requested capacity and the availability of competitive means of production which are cheaper, safer or cleaner in their application).

In a clinical chemistry laboratory, production takes place through a combination of different means of production (labour, raw materials and analysis instruments). Depreciation and interest over the capital invested are fixed costs. All the other costs which are necessary for the production are called *complementary costs* in the investment model presented here. The complementary costs include the exploitation costs, such as labour and raw materials, and the service costs of the investment. As a consequence, depreciation and interest, together with the complementary costs, form the total cost of the production.

### The investment model

In the investment model the criterion used to determine the optimal economic lifetime of a fixed production factor is chosen as the number of years in which the average cost price of the product is minimal. All the costs of the investment project are estimated in advance, so it is possible to calculate a price per product. In theory this price could also be used as the cost price.

The following assumptions are made in the application of this criterion:

(1) The expected development of the technique is nil (as soon as this changes strongly in reality, this development is immediately taken into account in the model and it is subsequently assumed that the development of the technique is constant).

(2) The present analysis process is continued at the same quantity of production for an indefinite period of time (as soon as the production quantities increase or decrease, this modification is incorporated in the model).

(3) The prices of the remaining production factors (such as labour and raw materials) stay unchanged.

It is only possible to determine the way in which depreciation of an analysis instrument takes place when its lifetime has come to an end. However, due to the fact that depreciation should be included in the cost price of laboratory investigations during use, it is necessary to make a prognosis of the way in which depreciation will occur as soon as the analysis instrument is put to use. If it appears that depreciation runs a different course during the period of use then the prognosis must be amended in the investment model.

As a result of the choice of a criterion: the same cost price for the whole period of use (as a guideline to specify the known costs), the basis for the depreciation plan in the investment model is determined by the condition that the sum of the complementary costs, depreciation and interest is constant assuming constant quantities of production. The choice did not fall on the potentially more applicable depreciation plan, in which the purchase price of the analytical instrument is divided over the expected period of use, taking the residual values into account (linear and book value depreciation systems). In the chosen depreciation method the same costs are calculated for equal performances regardless of the year in which these performances take place. The total costs of the analysis instrument (complementary costs, depreciation and interest) are divided over the number of years of use in proportion to the number of performances per year.

In practice it is common, after a certain period of use of an analytical instrument, for an alternative opportunity to arise where the same laboratory tests can be done for a lower cost price. This can now lead to a shortening or the end of the economic lifetime of the instrument which is being used – the value of the analytical instrument decreases or elapses due to technical developments. The moment at which the existing instrument should be replaced can be determined with the aid of the investment model.

The investment model is used to calculate the financial consequences of investment alternatives to support decision-making. It speaks for itself that in the question of purchase and replacement of analysis instruments other aspects must also be taken into consideration. Aspects such as the quality of clinical chemistry tests and of the provision of services, the influence of waste products on the environment and the ease of use and service grade are difficult to define in cost terms.

### The construction of the model

The first step in the construction of the model requires the following financial data to be collected:

(1) The sum to be invested (purchase price of the instrument, including installation and training costs).

2) The percentage of interest, to calculate the loss of investment opportunities.

(3) The variable exploitation costs per unit product (costs of chemicals and labour per test or request).

(4) The production quantities (the number of tests or requests per year) and the technical lifetime of the instrument. The remaining complementary costs per year, i.e. the fixed exploitation costs, must also be estimated. These include service and maintenance charges (labour and materials), and the costs of the loss of capacity due to breakdown (extra labour and raw materials). If necessary, these costs can be estimated *prospectively* on the basis of experience with similar instruments.

If it appears that estimates are not accurate after a certain period of use, the model must be refilled with data *retrospectively*. This may give rise to a different cost price, and, in some cases, a decision to replace the instrument.

In principle it is assumed that the residual value of the analytical instrument to be purchased is nil when the technical lifetime has elapsed. However, residual value can be taken into account in the investment model.

Costs which have been estimated at different times cannot be compared without further processing. They can be made comparable (and added together) after correction for the time difference, by calculating their *cash value*. In the investment model all expenses for complementary costs are made comparable to the expenses for the purchase of the instrument through conversion to the beginning of the first year of use (moment of purchase), according to the discounting method, in which the *annuity* is calculated. To do this it is necessary to know the amount of interest that will be lost.

The sum invested augmented by the cash value of the complementary costs of each year of use, results in the cash value of the total costs at the beginning of the investment. Due to the arithmetical assumption in the investment model that all products have been delivered at the end of each year, it is necessary to divide the total amount of costs equally over a number of years' use with the aid of the discounting method. The resulting amount, divided by the production quantities, results in the cost price per test or request. The application of the criterion of the minimal costs determines the economic lifetime.

In a clinical chemistry laboratory the products (i.e. test results) are delivered daily rather than annually. The investment model does not enter further into such specification, because, in essence, the results remain unchanged and consequently no extra information is supplied. In theory the model can be filled with data relating to a period of less than one year (and thus also to one day, for instance); for practical reasons this is, however, not very advisable for comparisons between potential purchases.

From the economic lifetime and the associated average costs per year calculated in this way, the system of depreciation can be determined. The basis of the system of depreciation is formed by the condition that the sum of the complementary costs, depreciation and interest is constant. This means that the average annual costs, less the complementary costs, result in the total amount available for interest and depreciation (to preserve the calculated minimal cost price). Depreciation is subsequently calculated by subtracting the interest over the book value of the instrument at the beginning of each year of use from the total amount available for interest and depreciation.

### An example of an investment

In this example the economic lifetime, depreciation and interest are calculated for a (fictitious) analytical instrument with the aid of the investment model.

It is assumed that the purchase price of an instrument is 15 000 Dutch guilders (Dfl.). The production quantity is 1000 tests per year. The technical lifetime is five years. The residual value of the instrument is nil. The interest is 10%.

*Table 1. Calculation of the economic lifetime of a fictitious analysis instrument on the basis of the criterion of minimal costs. All prices shown are in Dutch guilders. The lifetime in years is represented by* 'N'. (*See text for details.*)

| *N* | Total debit | Cash value complementary costs years 1 to 5 | Cash value total costs years 1 to 5 | Cost price per unit product per year |
|---|---|---|---|---|
| (1) | (2) | (3) | (4) | (5) |
| 1 | 15 000 | 10 909 | 25 909 | 28·50 |
| 2 | 15 000 | 21 653 | 36 653 | 21·12 |
| 3 | 15 000 | 32 547 | 47 547 | 19·12 |
| 4 | 15 000 | 44 158 | 59 158 | 18·66 |
| 5 | 15 000 | 56 577 | 71 577 | 18·88 |

The complementary costs, including the variable exploitation costs for a production of 1000 tests per year, in the successive years of use are considered to be made at the end of each year. The course is as follows:

Year 1 – Dfl. 12 000
Year 2 – Dfl. 13 000
Year 3 – Dfl. 14 500
Year 4 – Dfl. 17 000
Year 5 – Dfl. 20 000

The calculation for estimating the economic lifetime, based on the criterion of minimal costs, is shown in table 1.

In column 2 the total debit is shown for the use of the analysis instrument over one, two, three, four or five years. Since it is assumed that the residual value is nil, the purchase price is Dfl. 15 000 in each case.

In column 3 the complementary costs, which were made at different times, are discounted (for comparison and addition) by correcting the time difference. The expenses for the complementary costs should also be made comparable with the expenses for the purchase of the analytical instrument.

Therefore, all the costs mentioned above should be converted to the beginning of the first year (the moment of purchase). Discounting the complementary costs against an interest loss of 10% takes place as follows. The cash value of the complementary costs after various periods of use are:

One year Dfl. 12 000/1·10 = Dfl. 10 909
Two years Dfl. 10 909 + 13 000/1·10★1·10 = Dfl. 21 653
Three years Dfl. 21 653 + 14 500/1·10★1·10★1·10 = Dfl. 32 547
Four years Dfl. 32 547 + 17 000/1·10★1·10★1·10★1·10 = Dfl. 44 158
Five years Dfl. 44 158 + 20 000/1·10★1·10★1·10★1·10★1·10★ = Dfl. 56 577

In column 4 the total costs from a period of use of one to five years are obtained by adding the cash value of the complementary costs (column 3) to the purchase price

(column 2). In the investment model the arithmetic assumption is made that the products have been delivered by the end of the year. If the analysis instrument is used for a period of one year, then the total amount – Dfl. 25 909 – has to be carried to the end of the year (multiplied by the interest loss of 10%). If the analysis instrument is used for a period of two years, then the total amount – Dfl. 36 653 – must be equally divided over two years with the aid of the calculation of annuity:

Cash value of the total costs ★ Ann[*n*]10 = Cash value of the annual costs.

The annuity after *n* years of use with an interest of 10% is represented by the annotation Ann[*n*]10 obtained from annuity tables. The amounts shown in column 4 are converted into the cash value of the annual costs as follows. After a period of use of:

| | | | | |
|---|---|---|---|---|
| One year | Dfl. 25 909 | ★ | 1.100000 = | Dfl. 28 500 |
| Two years | Dfl. 36 653 | ★ | 0.576190 = | Dfl. 21 119 |
| Three years | Dfl. 47 547 | ★ | 0.402115 = | Dfl. 19 119 |
| Four years | Dfl. 59 158 | ★ | 0.315471 = | Dfl. 18 663 |
| Five years | Dfl. 71 577 | ★ | 0.263797 = | Dfl. 18 882 |

The cost prices per unit product (test or request) shown in column 5 are subsequently obtained by dividing by the production quantities (1000 units per year).

In this example the cost price appears to be minimal with a period of use of four years. From the average annual costs related to this the debits (see table 2), can be calculated. The total costs per year amount to Dfl. 18 663 and are shown in column 2. The annual costs, less the complementary costs from column 3, result in the total amount available for interest and depreciation and are given in column 4. Finally, the latter amount less the interest (10%) over the book value at the beginning of the relevant year from column 5, results in the depreciation in that year, as shown in column 6.

This example may seem rather complicated, but is easy to adapt. In the first place the residual value of an analytical instrument is not usually nil at the end of its economic lifetime (the machine can often be traded-in). In this calculation the residual value can be taken up by applying the difference between the purchase price and cash price of the residual value at the end of the relevant year instead of the total purchase price (see table 1, column 2).

Secondly, there is a possibility that during the use of an analytical instrument an alternative instrument, which can carry out clinical chemistry tests for a lower price, will come onto the market.

This will reduce the economic lifetime of the analytical instrument purchased – the existing instrument having been replaced. The most suitable moment to replace the instrument – with regard to economics – can be calculated with the investment model.

Thirdly, there are usually several alternative machines available and the model can be used to compare the various options.

Assuming that two years after the analytical instrument has been put to use, a new instrument becomes available which is capable of achieving the desired production quantities of 1000 units per year for a total amount of Dfl. 16 000, then the cost price per unit is Dfl. 16·00 with the new instrument as against Dfl. 18·66 with the existing instrument.

The complementary costs in the fourth year of use of the original instrument are Dfl. 17 000; the value of the activities is, however, Dfl. 16 000 (it is possible to carry out clinical chemistry tests for this price using the new instrument). Therefore, the value of the activities of the existing analysis instrument is negative (in this case minus Dfl. 1000). If the same reasoning is applied to the third year of use, it appears that the performance of the existing instrument is positive (in this case Dfl. 16 000–Dfl. 14 500 = Dfl. 1500). As it is still assumed that the residual value is nil, it would seem to be worthwhile from an economic point of view, to replace the existing instrument with the alternative instrument at the end of its third year of use. By doing this there is a loss of book value of Dfl. 1511 (the part not yet debited). Through the availability of a more economically worthwhile alternative, the economic lifetime of the existing instrument has decreased from four to three years.

### Application of the model

The calculations in the investment model can be carried out with the aid of a microcomputer running a spreadsheet program. The application program is based on Lotus 1-2-3 and is suitable for use on an IBM-compatible microcomputer with an internal memory of at least 384 kB, MS/DOS operating system.

*Table 2. Calculation of the debits and interest in the case of a known economic lifetime and associated annual costs. All prices shown are in Dutch guilders. (See text for details.)*

| Year (1) | Annual costs (2) | Complementary costs (3) | Depreciation and interest (4) | Interest (5) | Debit (% of purchase price) (6) |
|---|---|---|---|---|---|
| 1 | 18 663 | 12 000 | 6663 | 1500 | 5163 (34·4%) |
| 2 | 18 663 | 13 000 | 5663 | 984 | 4679 (31·2%) |
| 3 | 18 663 | 14 500 | 4163 | 516 | 3647 (24·3%) |
| 4 | 18 663 | 17 000 | 1663 | 152 | 1511 (10·1%) |
| Total | 74 652 | 56 500 | 18 152 | 3152 | 15 000 (100·0%) |

### Acknowledgements

The help of H. Kamps and B. J. M. Hanewinkel in creating this investment model is greatly appreciated by the authors.

### References

1. Broughton, P. M. C. and Hogan, T. C., *Annals of Clinical Biochemistry,* **18** (1981), 330–342.
2. Worth, H. G. L., *Journal of Automatic Chemistry,* **2** (1980), 125–133.
3. Gibitz, H. J., *Journal of Automatic Chemistry,* **5** (1983), 79–82.
4. Leijten, J. F., van der Geer, F., Scholten, M. N. M. and Goldschmidt, H. M. J., *Annals of Clinical Biochemistry,* **21** (1984), 109–115.
5. Hindriks, F. R., van der Slik, W., Bosman, A., Frowein, Chr. and Kamps, H., *Journal of Automatic Chemistry,* **8** (1986) 178–185.
6. Bouma, J. L., *De theorie van de financiering van ondernemingen.* Leerboek der bedrijfseconomie, part 2 (Delwel, Wassenaar, The Netherlands, 1980).
7. Bouma, J. L., Nijenhuis, W. A. and van de Poel, J. H. R. Syllabus management accounting, part 1. (University of Groningen, Economic Faculty, Groningen, The Netherlands, 1984).

### Glossary

*Annuity*

Average sum per year (using one currency), in which the capital value of an investment project can be deduced through the calculation of the market value.

*Book value*

The value of a means of production according to the financial administration at the end of a year's use.

*Complementary costs*

All costs, apart from depreciation and interest costs, which can be directly attributed to the performance of an analysis instrument which is in production.

*Economic lifetime*

The length of time that it is economically worthwhile to use an instrument.

*Fixed assets*

Capital purchases from which the application is attributed to successive production processes. The plusses and minusses are divided over the successive production processes and usually over several financial years. Fixed assets are often called 'long-lasting means of production'.

*Floating assets*

Capital purchases, in contrast to fixed assets, in which investments are made for only short periods – not more than one year. Floating assets are often called 'floating means of production'.

*Investment project*

The total investment in long-lasting means of production, with all additional investments in floating means of production.

*Market (cash) value*

Costs which have been reduced from the time they were made to an earlier point in time (discounting).

*Residual value*

The value of a means of production at the end of its last year of use.

*Technical lifetime*

The length of time that an instrument is technically capable of performing the activities for which it was purchased.

